# First Draft Genome Sequence of the Pathogenic Fungus *Lomentospora prolificans* (Formerly *Scedosporium prolificans*)

**DOI:** 10.1534/g3.117.300107

**Published:** 2017-09-29

**Authors:** Ruibang Luo, Aleksey Zimin, Rachael Workman, Yunfan Fan, Geo Pertea, Nina Grossman, Maggie P. Wear, Bei Jia, Heather Miller, Arturo Casadevall, Winston Timp, Sean X. Zhang, Steven L. Salzberg

**Affiliations:** * Center for Computational Biology, McKusick-Nathans Institute of Genetic Medicine, Johns Hopkins University School of Medicine, Baltimore, Maryland 21205; † Department of Computer Science, Johns Hopkins University, Baltimore, Maryland 21218; ‡ Institute for Physical Sciences and Technology, University of Maryland, College Park, Maryland 20742; § Department of Biomedical Engineering, Johns Hopkins University School of Medicine, Baltimore, Maryland 21205; ** Department of Molecular Microbiology and Immunology, Bloomberg School of Public Health, Johns Hopkins University, Baltimore, Maryland 21205; †† Department of Pathology, Johns Hopkins University School of Medicine, Baltimore, Maryland 21287; ‡‡ Microbiology Laboratory, Johns Hopkins Hospital, Baltimore, Maryland 21287; §§ Department of Biostatistics, Bloomberg School of Public Health, Johns Hopkins University, Baltimore, Maryland 21205

**Keywords:** genome assembly, fungal genomics, nanopore sequencing, pathogen genomics

## Abstract

Here we describe the sequencing and assembly of the pathogenic fungus *Lomentospora prolificans* using a combination of short, highly accurate Illumina reads and additional coverage in very long Oxford Nanopore reads. The resulting assembly is highly contiguous, containing a total of 37,627,092 bp with over 98% of the sequence in just 26 scaffolds. Annotation identified 8896 protein-coding genes. Pulsed-field gel analysis suggests that this organism contains at least 7 and possibly 11 chromosomes, the two longest of which have sizes corresponding closely to the sizes of the longest scaffolds, at 6.6 and 5.7 Mb.

*Lomentospora prolificans* is an opportunistic fungal pathogen that causes a wide variety of infections in immunocompromised and immunocompetent people and animals (Rodriguez-Tudela *et al.* 2009; Cortez *et al.* 2008). It was originally proposed as *Scedosporium inflatum* in 1984 by Malloch and Salkin (Salkin *et al.* 1988), and later renamed as *Scedosporium prolificans* in 1991 by Geuho and de Hoog (1991). In 2014, the fungus was renamed *Lomentospora prolificans* based on its phylogenetic distance from other *Scedosporium* species (Lackner *et al.* 2014a).

*L. prolificans* is distributed throughout the world and primarily found in soil and plants. Transmission to humans is often via inhalation of the spores produced by the fungus, but occasionally it is introduced by direct traumatic inoculation. Infections can range from mild, local infections to severe and disseminated, with the latter being life-threatening. Invasive infections have been increasingly associated with hematological malignancies, transplantation, and cystic fibrosis (Rodriguez-Tudela *et al.* 2009). The major challenge to successful therapy is that the fungus is intrinsically resistant to almost all antifungal drugs currently available for treatment (Lackner *et al.* 2014b). As a result, the clinical outcome of invasive infections is often fatal.

## Materials and Methods

### Isolation and growth

The *L. prolificans* strain included in this study was isolated from a bronchoalveolar lavage fluid sample of a cystic fibrosis patient, in whom it was causing a chronic refractory pulmonary fungal infection. The fungus was cultivated on Potato Flake Agar plates (BD, Sparks, MD) at 30° until reaching sizable colonies with adequate sporulation. Spores were collected and then converted into a hyphal mass by growing in Sabouraud Liquid Broth (BD, Sparks, MD) under a 20-rpm rotator (Stuart, Staffordshire, UK) at 23°. Genomic DNA was extracted from the hyphal mass by using ZR Fungal/Bacterial DNA MiniPrep kits (Zymo Research, Irvine, CA) according to the manufacturer’s protocol with the following modification: a horizontal vortex adapter (Mo Bio, Carlsbad, CA) was used with the 10-min beads beating step during the cell lysis step.

### Illumina sequencing library construction

Nextera XT (Illumina) transposase-based libraries were generated with 1 ng of purified, unsheared *L. prolificans* hyphal DNA. After transposition and barcoded adapter ligation by PCR, the library was purified using 0.4× AMPure XP (Beckman Coulter) and size profiles generated using the Agilent Bioanalyzer high-sensitivity chips, average size 747 bp. The library was normalized to 4 nM, then paired-end, dual index sequencing was performed using Miseq v2 500 cycle chemistry.

### Nanopore sequencing library construction and data preparation

We input 1.5 μg purified, unsheared *L. prolificans* hyphal DNA into the LSK-108 Oxford Nanopore Technologies (ONT) ligation protocol. The library was blunt-ended and A-tailed with NEB Ultra II End prep module, and purified using 1× AMpure XP. Adapter ligation was then performed using Blunt-TA ligase master mix (NEB) and proprietary ONT “1D” adapters containing a preloaded motor protein. The library was purified using 0.4× Ampure XP, washed with buffer WB (ONT), and eluted with elution buffer EB (ONT), which contains a tether molecule that directs library molecules toward the nanopore membrane surface. The library was sized using the Agilent Bioanalyzer high-sensitivity chips, size peaking at 2.8 kb. The entire library was mixed with running buffer and sepharose loading beads (ONT), then run on a R9.4 SpotON MinION flowcell for 48 hr. Raw fast5 files were basecalled using Albacore version 1.0.2, and fastq files were extracted using our custom python script.

### Sequencing

The Illumina MiSeq run generated 12.04 million 250-bp paired-end reads with a mean fragment size of 500 bp, for a total of 6.02 Gbp of data. The Oxford Nanopore MinION run produced ∼3.66 million reads for a total of 4.3 Gbp of data. Among the MinION reads, 1.18 million reads (2.96 Gbp) were longer than 1 kbp, and 25,788 reads (333.27 Mbp) were longer than 10 kbp.

### Pulsed-field gel analysis

Conidia were inoculated into Sabouraud dextrose broth, grown with shaking at 30° for 2–3 d, and used to generate protoplasts using minor modifications of published methodology (Al-Laaeiby *et al.* 2016). Briefly, fungal biomass was filtered through a cell strainer, washed with sterile water and incubated at 30° on a nutating mixer for 3 hr in OM buffer with 5% Glucanex. Contents were then split into sterile centrifuge tubes and overlaid with chilled ST buffer in a ratio of 1.2 ml fungal solution to 1 ml ST buffer. Tubes were then centrifuged at 5000 × *g* for 15 min at 4°. Protoplasts were recovered at the interface of the two buffers and transferred to a sterile centrifuge tube, to which an equal volume of chilled STC buffer was added. Protoplasts were pelleted at 3000 g for 10 min at 4°, following which supernatant was removed, and protoplasts were resuspended in 10 ml STC buffer. This was repeated two more times, with the final resuspension being performed with 200 μl GMB buffer. Plugs were then made and treated using methodology adapted from Brody and Carbon (1989). Briefly, 200 μl 1–2 × 10^9^ protoplasts in GMB buffer were mixed with 200 μl 2% low-melt agarose in 50 mM EDTA (pH 8) cooled to 42° and pipetted into plug molds, which were then placed on ice for 10 min to solidify. Plugs were removed from their molds and incubated in NDS buffer with proteinase K at 50° for 24 hr, followed by three 30-min washes in 50 mM EDTA (pH 8) at 50°. Plugs were stored in 50 mM EDTA (pH 8) at 4°.

Plugs were inserted into agarose gels, along with *S. cerevisiae* and *S. pombe* size standards (Bio-Rad Laboratories, Hercules, CA) and clamped homogeneous electrical field (CHEF) electrophoresis was run on a CHEF-DR III (Bio-Rad Laboratories). The gel showing the full range of bands was captured using the conditions described in the CHEF-DR III manual for *Hansenula wingei* with the following exception: gel was made from 0.8% SeaKem Gold agarose (Lonza, Basel, Switzerland). The gel showing the smaller bands in greater resolution was made using 0.6% SeaKem Gold agarose in 0.5× TBE, with one block of 4.5 V/cm at an included angle of 120° and switch time of 60–300 sec for 24 hr, followed by a second block of 2.0 V/cm at an included angle of 106° and switch time of 720–900 sec for 12 hr. The run was conducted at 12°.

### Data availability

This genome project has been deposited at NCBI/GenBank as BioProject PRJNA392827, which includes the raw read data, assembly, and annotation. The assembly is available under accession NLAX00000000; the version described in this paper is version NLAX01000000. The assembly and annotation is also available from the authors’ ftp site at ftp.ccb.jhu.edu/pub/data/assembly/L_prolificans.

## Results and Discussion

### Assembly

**Figure 1:**
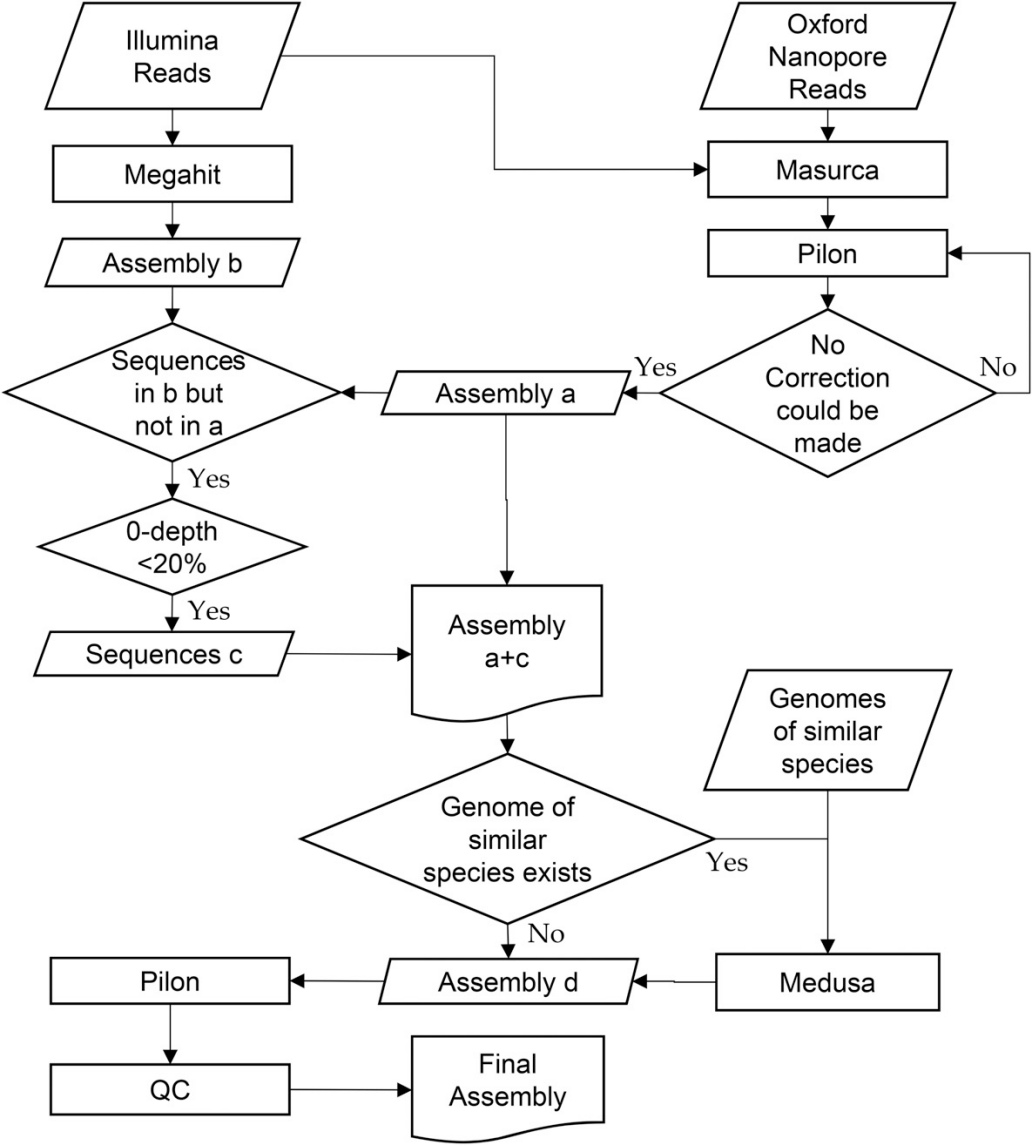
Genome assembly pipeline used for hybrid assembly of *L. prolificans* from Oxford Nanopore and Illumina reads. Both data sets were assembled jointly with MaSuRCA, and Illumina reads were assembled separately with Megahit, followed by assembly polishing, comparison, and merging steps. The genomes of two related *Scedosporium* species, *S. apiospermum* and *S. aurantiasum*, were used to improve scaffolding.

We used multiple tools in our assembly pipeline (Figure 1). First, we used the MaSuRCA genome assembler (Bosi *et al.* 2015) (version 3.2.2_RC3) to assemble both types of data, with default settings except for the option “+USE_LINKING_MATES=1.” Then we used the Megahit genome assembler (Li *et al.* 2015) (version 1.1.1) to assemble the Illumina data separately. We then aligned the Megahit assembly to the MaSuRCA assembly using NUCmer (version 3.1) (Kurtz *et al.* 2004), and found 2599 sequences in the Megahit assembly, summing up to 1.01 Mbp, that were missing in the MaSuRCA assembly. We added these sequences to the MaSuRCA assembly to form a more complete set of contigs.

Next, we used the MeDuSa scaffolder (version 1.6) (Bosi *et al.* 2015) to determine the correct order and orientation of the contigs using the assemblies of two related organisms *S. apiospermum* (Vandeputte *et al.* 2014) and *S. aurantiasum* (Perez-Bercoff *et al.* 2015). The resulting scaffolds were polished and gap-filled by Pilon (version 1.5) (Walker *et al.* 2014) iteratively for 15 rounds until no additional correction could be made. Note that both Illumina reads and MinION reads were used in the first round of polishing, but only Illumina reads were used in the last 14 rounds. Then we aligned the polished scaffolds against the Univec and the Emvec database to ensure no vector sequence was contained in the assembly. Finally, we aligned the Illumina reads and Nanopore reads to the scaffolds using the BWA aligner (version 0.7.15) (Li 2013). Using these alignments, we broke apart scaffolds at positions with zero physical coverage (*i.e.*, no read coverage and no read pairs spanning a position), except for the leading and trailing 100 bp.

**Figure 2:**
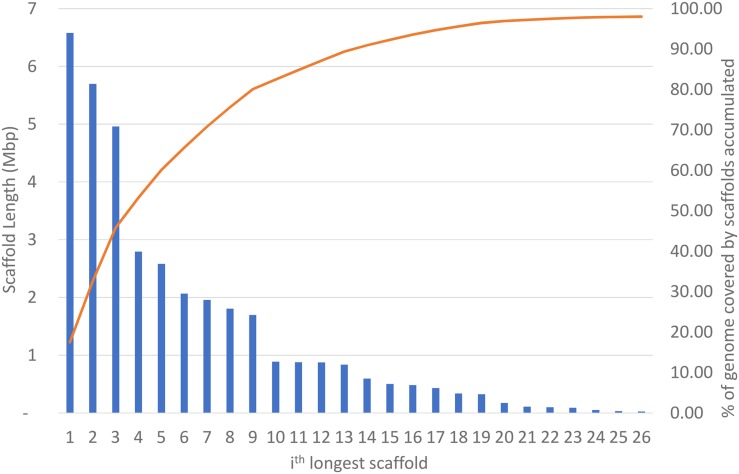
The sizes of the 26 longest scaffolds (blue bars, size shown on left) and the cumulative percentage of the total assembly that they comprise (red line, percentage shown on right).

The final assembly consists of 1625 scaffolds (240 to 6,579,848 bp in length) and has a total size of 37,627,092 bp with 51.46% GC content. The N50 size is 2,796,173 bp. The longest 26 sequences comprise 98.02% of the assembly (Figure 2). Four of the longest scaffolds (lengths 6.6 Mb, 1.8 Mb, 876 Kb, and 837 Kb) contain telomeric repeats on one end. The mitochondrial sequence assembled into a single contig, which upon closer inspection had bases on both ends that overlapped, confirming that it was circular. The redundant bases were trimmed in the final assembly, and the final mitochondrion (the 27th largest scaffold, renamed “mitoscaff1”) contains 23,987 bp and 12 protein-coding genes.

Supplemental Material, Figure S1 and Figure S2 in File S1 show comparisons of preliminary assemblies using Illumina data and Nanopore data separately, and using different assembly programs [Megahit (Li *et al.* 2015), SPAdes (Bankevich *et al.* 2012), SSPACE (Boetzer *et al.* 2011), and MaSuRCA (Bosi *et al.* 2015)]. The figures demonstrate that the Illumina-only assembly is more complete although far more fragmented than the Nanopore-only assembly. The hybrid assembly combines the benefits of both approaches, producing longer scaffolds and a more complete genome.

**Figure 3:**
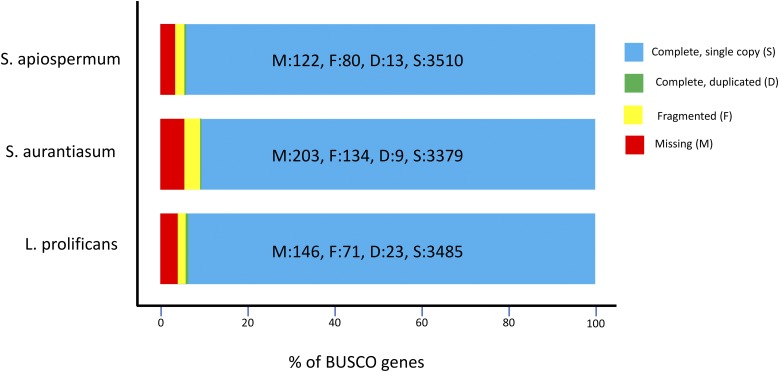
Results from searching for a set of conserved, single-copy genes in *L. prolificans* (bottom) and its two closest sequenced relatives, *S. apiospermum* and *S. aurantiasum*.

We used the BUSCO pipeline (v3) (Simao *et al.* 2015) to assess the genome assembly completeness. BUSCO searches for the presence of genes that occur as single-copy orthologs in at least 90% of a lineage. We used the lineage “Sordariomycetes,” which contains 3725 orthologous groups and is the class containing *L. prolificans*. Our *L. prolificans* assembly covers 94.2% of the groups, while the *S. apiospermum* assembly and the *S. aurantiasum* assembly cover 94.5 and 90.9% of groups, respectively (Figure 3).

### Annotation

We used the MAKER automated annotation system (Campbell *et al.* 2014) to identify protein-coding genes in the assembly. The primary evidence for annotation was protein and EST alignments from other fungi, which identified 8539 genes, of which 7477 were multi-exon transcripts. We then trained three *ab initio* gene finders (Augustus, SNAP, and GeneMarkES) and provided their outputs to MAKER in a second pass. MAKER uses these predictions to modify the alignment-based gene models, although it does not predict any genes based solely on *ab initio* predictions.

After the second pass of annotation, we identified 8896 putative transcripts of which 7117 contain >1 exon. (Currently there are no alternative splice variants, thus there is a 1:1 ratio between transcripts and genes.) These transcripts covered 13,404,230 bp (35.62% of the genome), of which 13,299,472 bp are protein-coding and the remainder are 3′ and 5′ untranslated regions. By comparison, the annotation of the draft genomes of *S. apiospermum* and *S. aurantiasum* contain 10,919 and 10,525 genes, respectively. All of these gene predictions are based on automated annotation pipelines and should be regarded as preliminary. A phylogenetic tree showing the relationship of these three species to neighboring Ascomycete fungi is shown in Figure S3 in File S1.

### Chromosome structure

**Figure 4:**
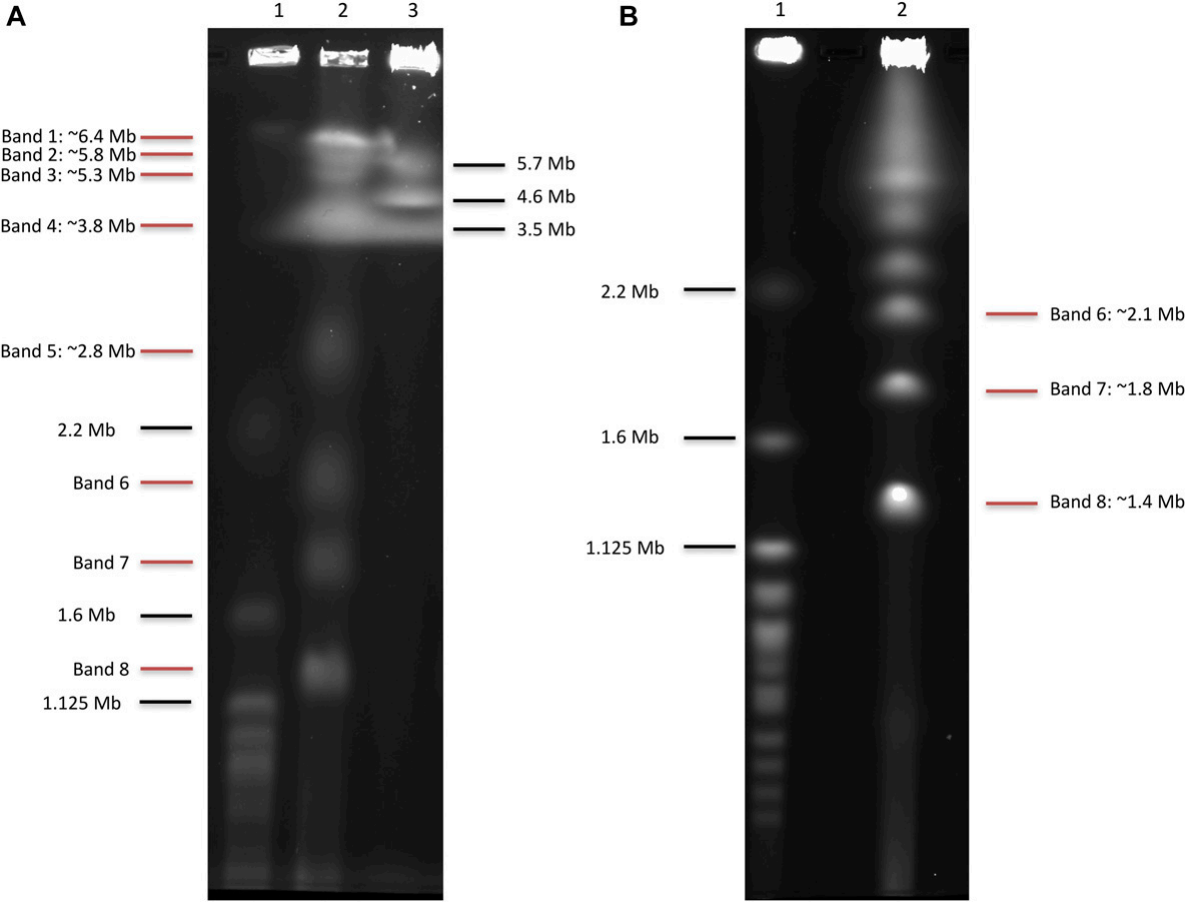
Separation of *L. prolificans* chromosomal DNA by PFGE. (A) Chromosomal DNA from *S. cerevisiae* (lane 1), *L. prolificans* (lane 2), and *S. pombe* (lane 3) were run, allowing estimation of the number and lengths of the *L. prolificans* chromosomes, as indicated. (B) Chromosomal DNA from *S. cerevisiae* (lane 1) and *L. prolificans* (lane 2) were separated under conditions optimized for chromosomes in the range of 0.9–3.2 Mb, allowing more precise estimates of lengths of chromosomes contained in *L. prolificans* bands 6–8.

To determine if the assembly was consistent with laboratory estimates of genome size, we separated and visualized the *L. prolificans* chromosomes in agarose by pulsed-field gel electrophoresis (PFGE). While not all chromosomes appeared fully resolved, the lane with the best resolution showed bands for at least eight chromosomes, at between ∼1.4 and 6.4 Mb in length (Figure 4A), relative to *S. cerevisiae* and *S. pombe* standards. The chromosome banding pattern above 3.5 Mb was not consistently observed between two sample lanes (data not shown), which may indicate these larger chromosomes were not stable and/or degraded quickly. Of the observed bands, three show staining consistent with multiple chromosomes of the same approximate size migrating together (Figure 4A, bands 1, 4, and 8). It should be noted that the largest two bands were larger than the largest *S. pombe* chromosome, decreasing our ability to predict their sizes.

A second PFGE (Figure 4B) performed under conditions designed to better resolve bands in the range of 0.9–3.2 Mb allowed us to more precisely estimate the sizes of the three smallest bands, which we estimate to be 1.4, 1.8, and 2.1 Mb, respectively. The 1.4-Mb band (band 8) showed staining consistent with two chromosomes migrating together. From these gels we can determine that *L. prolificans* contains at least 7, and likely 11, chromosomes. We observe staining consistent with at least two chromosomes at sizes 1.4, ∼3.8, and ∼6.4 Mb. These three sets of two chromosomes, along with those within the range of the standards, 1.8, 2.1, 2.8, 3.8, and 5.3 Mb, plus a single band which is outside of the range, but estimated ∼5.8 Mb, sum up to 41 Mb. Given the uncertainty of these estimates, the total approximate genome size is consistent with our 37.6-Mb genome assembly. The two largest scaffolds (Figure 2) have lengths 6.6 and 5.7 Mb, which is in agreement with the PFGE results for the largest chromosomes.

## Supplementary Material

Supplemental material is available online at www.g3journal.org/lookup/suppl/doi:10.1534/g3.117.300107/-/DC1.
