# Supplementary material for: First Draft Genome Sequence of the Pathogenic Fungus Lomentospora prolificans (Formerly Scedosporium prolificans)
Source: G3 (Bethesda). 2017 Sep 29;7(11):3831–6. doi: 10.1534/g3.117.300107 (PMC5677167; doi:10.1534/g3.117.300107)
Supplement: Supplementary file 1 [file 3831FileS1.docx]

*
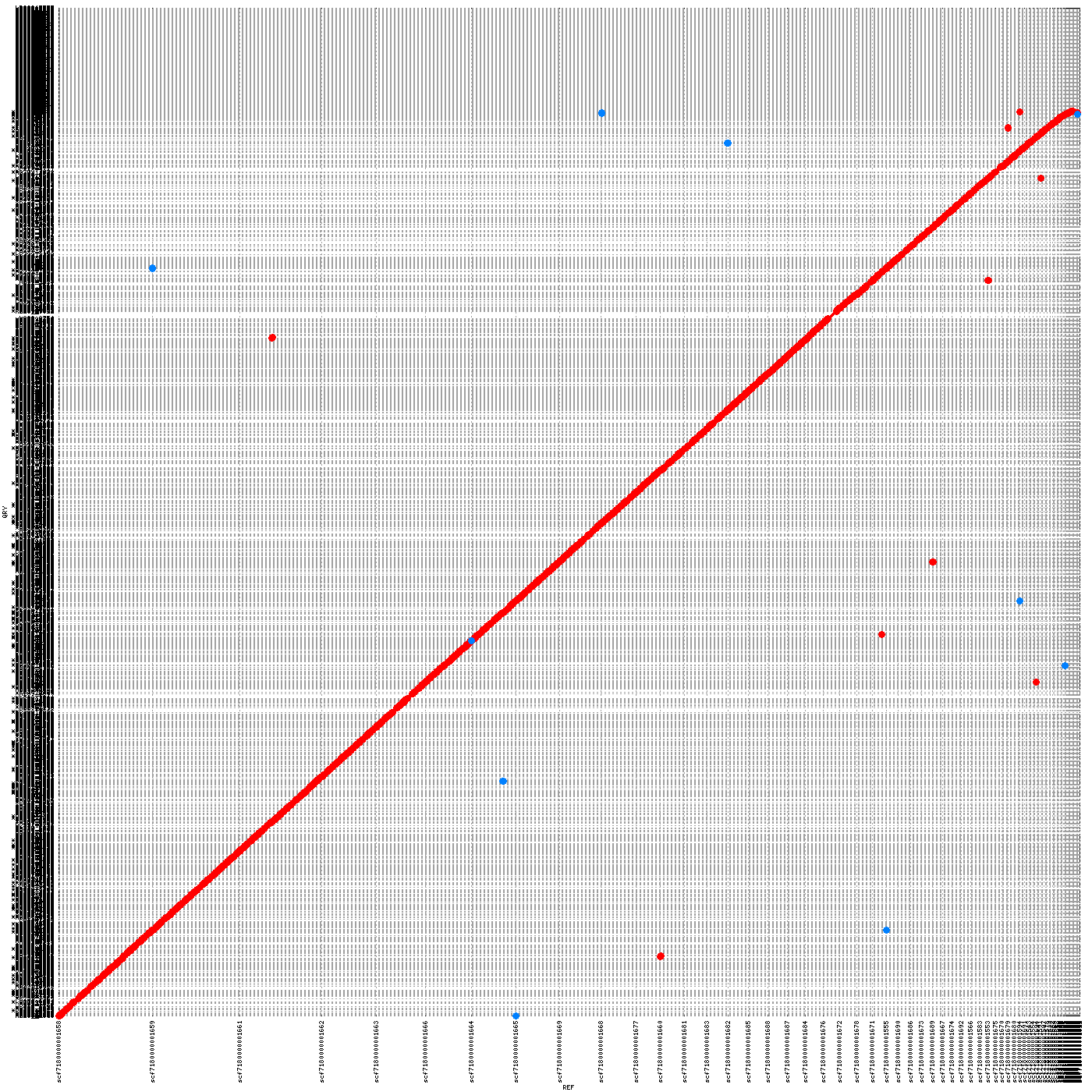
*

**Supplementary figure 1**. Dot plot alignment of the Illumina-only assembly (y-axis) versus the Nanopore-only assembly (x-axis). The Illumina-only assembly used the MEGAHIT (Li et al., 2015) and SSPACE (Boetzer et al., 2010) assemblers for contigging and scaffolding respectively, 11,785 scaffolds were generated producing a scaffold N50 of 34,127 bp, approximately two orders of magnitude shorter than our final assembly. A large number of Illumina-based contigs, shown in the upper portion of plot, fail to align to any of the Nanopore-only contigs, demonstrating that the Nanopore-only assembly was less complete. The Illumina contigs were aligned to the NCBI Reference database (Sept 2015 version) to confirm that they were not contaminants.

**Supplementary figure 2**. A comparison of weighted average scaffold sizes for four different assembly strategies. The strategies include using the Megahit with Illumina data, using the SPAdes assembler with Illumina data, using SPAdes with Illumina plus Nanopore data, and using the approach we adopted, MaSuRCA + Megahit on both sets of data. The N(X) size is shown on the y-axis, where N(X) is defined as the contig/scaffold size such that X% of the genome is contained in contigs/scaffolds equal to or larger than the N(X) length. Genome size was set to 40Mbp for all four plots shown here. Thus N50 size is the size of the smallest scaffold such that 50% of the genome is contained in scaffolds of that size or larger. The MaSuRCA+Megahit strategy consistently produced the largest scaffolds.

**Supplementary figure 3**. Evolutionary relationship of *Lomentospora prolificans* with other fungal species. The tree was built using sequences from each genomes' internal transcribed spacer (ITS) region. Sequences were obtained from the following GenBank accessions: JQ906772.1 (*Beauveria bassiana*), KM231797.1 (*Fusarium solani*), LM652465.1 (*Microascus brevicaulis*), KF686759.1 (*Pseudogymnoascus destructans*), NR_130684 (*Sarocladium kiliense*), AJ888416.1 (*Scedosporium apiospermum*), AJ888440.1 (*Scedosporium aurantiacum*), KX590842.1 (*Sporothrix schenckii*). The sequences were aligned using Muscle^1^, and the multiple alignment is shown in Supplementary Figure 4. The tree was built in MEGA6 ^2^ using the neighbor-joining method. The percentage of replicate trees in which the associated taxa clustered together in the bootstrap test (1000 replicates) are shown next to the branches. The tree is drawn to scale, with branch lengths in the same units as those of the evolutionary distances used to infer the phylogenetic tree.

Beauveria_bassiana_ATCC_MYA_4886 TACCG-AGTTTTC-------------AACTCCCTA-----------ACCCTTCTGTGAAC

Fusarium_solani_CBS_119600 TACCG-AGTTATT-----------CA--ACTCATC-----------AACC-C-TGTGAAC

Lomentospora_prolificans_JHH_5317 TACCG-AGTTATT---------------ACTCCAA-----------ACCCTT-TGTGAAC

Microascus_brevicaulis_MUCL_40726 TACCGAAGTTACT---------------CTTCAAA-----------ACCCAT-TGTGAAC

Pseudogymnoascus_destructans_ATCC TACAGTAGTCGCCCGGGTTGCCGCAAGGCCTCCCGGGTAACCTACCACCCTT-TGTTTAT

Sarocladium_kiliense_MUCL_9724 TACCAGAGTGCCC-----------TAGGCTCTCCA-----------ACCCAT-TGTGAAC

Scedosporium_apiospermum_FMR_8619 TACAG-AGTTACT---------------ACTCC-A-----------ACCCAT-TGTGAAC

Scedosporium_aurantiacum_FMR_8630 TACAG-AGTTACT---------------ACTCCAA-----------ACCCAT-TGTGAAC

Sporothrix_schenckii_CBS_359.36 TACAG-AGTTTTC----------ACA--ACTCCCA-----------ACCC-T-TGCGAAC

Beauveria_bassiana_ATCC_MYA_4886 C-TACCT-AT--------CGTTGCTTC-GGCGG--------------ACTCGCCCCAGCC

Fusarium_solani_CBS_119600 A-TACCTAAA--------CGTTGCTTC-GGCGGGAAC----------AGACGGC---CCC

Lomentospora_prolificans_JHH_5317 CTTACCT-ATGTT-C---TGTTGCCTC-GGCGG-GGA----------GGAAGAC---CCC

Microascus_brevicaulis_MUCL_40726 CTTACCTCTTGCCGCG--CGTTGCCTC-GGCGGGGAGGCGGGGTCTGGGTCGGC---GCG

Pseudogymnoascus_destructans_ATCC TACACTT-----------TGTTGCTTT-GGCA---------------GGCCTGC---CCT

Sarocladium_kiliense_MUCL_9724 A-TACCT-AT--------CGTTCCCTC-GGCGG--------------GCTCAGC------

Scedosporium_apiospermum_FMR_8619 CTTACCT-ATGTT-C---TGTTGCCTC-GGCGGCGTC----------GGTCAGC---GCC

Scedosporium_aurantiacum_FMR_8630 CTTACCT-ATGTT-C---TGTTGCCTC-GGCGG-GGC----------GGTCGGC---GCC

Sporothrix_schenckii_CBS_359.36 CGTACCCAATCTCGTTCTCGTTGCTTCTGGCGGGGGG---GGGAGCGGGGGGGC---GCC

Beauveria_bassiana_ATCC_MYA_4886 CGGACGCGGACTGGACCAGCG----------------GCCCGCC-GGGGACCTCAAACTC

Fusarium_solani_CBS_119600 G-----TGAAACGGGCCGC------------------CCCCGCCAGAGGACCCCTAACTC

Lomentospora_prolificans_JHH_5317 T------TAAAAAGGGCCCAG---------C------CCCCGCC-GGCAGCACCAAACTC

Microascus_brevicaulis_MUCL_40726 C---CCCTCACCGGGCCGCCGTCCCCGTCCCCGT---CCCCGCC-GGCCGCGCCAAACTC

Pseudogymnoascus_destructans_ATCC C-----------GGGCTGCTGGCTCCGGCCGGCGAGCGCTTGCC--AGAGGACTAAACTC

Sarocladium_kiliense_MUCL_9724 -------------GCGCGGTGCCTCCGGGCTCCGGGCGTCCGCCGGGGACAACCAAACTC

Scedosporium_apiospermum_FMR_8619 C---CTCTGAGAAGAG----GACGATGCCCC------TCCCGCC-GGCAGCACCAAACTC

Scedosporium_aurantiacum_FMR_8630 CCCTCTCCGCGAGGAGGGGAGACGATGGACC------CCCCGCC-GGCAGCACCAAACTC

Sporothrix_schenckii_CBS_359.36 C-----------GACACGGCCCCCTCCGCCCCC----GCCCGCC-AGGGGCGGCGGGCCC

Beauveria_bassiana_ATCC_MYA_4886 TTGTATTCC------------AGCATCTTCTGA-ATACGCCGCAAGGCAAAACAAATG--

Fusarium_solani_CBS_119600 T--GTTTCT---ATAATGTTTC-----TTCTGA-GTAAAACA--------AGCAAATA--

Lomentospora_prolificans_JHH_5317 TTGCA-TTT---ATAGCGGATTAC-ATTTCTGA-ATACAATAC---------AAAACA--

Microascus_brevicaulis_MUCL_40726 T--AAATTTGAAAAAGCGTACTGCACGTTCTGA-TT-CAAAAC-------AAAAAACA--

Pseudogymnoascus_destructans_ATCC TGTTTGTCT---ATACTG----------TCTGA-GTACTATAT-----------AAT---

Sarocladium_kiliense_MUCL_9724 T--GATTTT---ATTGTGAATC------TCTGAGGGGCGAAAGCCCGAAAACAAAATG--

Scedosporium_apiospermum_FMR_8619 TTGAATTTT---ACAGCGGATTAC-AGTTCTGA-TTTGAAAAC-------AAAAAACA--

Scedosporium_aurantiacum_FMR_8630 TTGAATTTT---ATAGCGGATTACAAGCTCTGA-TTGGAAAAC------AAAAAAACA--

Sporothrix_schenckii_CBS_359.36 TACGAACCT------TTGTATCTCAACCACTAG-AAAAACCGT-CTGAGGAAAAAACAAA

Beauveria_bassiana_ATCC_MYA_4886 --AATCAAAACTTTCAACAACGGATCTCTTGGCTCTGGCATCGATGAAGAACGCAGCGAA

Fusarium_solani_CBS_119600 --AATTAAAACTTTCAACAACGGATCTCTTGGCTCTGGCATCGATGAAAAACGCACCGAA

Lomentospora_prolificans_JHH_5317 --AATCAAAACTTTCAACAACGGATCTCTTGGTTCTGGCATCGATGAAGAACGCAGCGAA

Microascus_brevicaulis_MUCL_40726 --AGTCAAAACTTTTAACAACGGATCTCTTGGTTCTGGCATCGATGAAGAACGCAGCGAA

Pseudogymnoascus_destructans_ATCC --AGTTAAAACTTTCAACAACGGATCTCTTGGTTCTGGCATCGATGAAGAACGCAGCGAA

Sarocladium_kiliense_MUCL_9724 --AATCAAAACTTTCAACAACGGATCTCTTGGCTCTGGCATCGATGAAGAACGCAGCGAA

Scedosporium_apiospermum_FMR_8619 --AGTTAAAACTTTCAACAACGGATCTCTTGGTTCTGGCATCGATGAAGAACGCAGCGAA

Scedosporium_aurantiacum_FMR_8630 --AATCAAAACTTTCAACAACGGATCTCTTGGTTCTGGCATCGATGAAGAACGCAGCGAA

Sporothrix_schenckii_CBS_359.36 ATAATCAAAACTTTCAACAACGGATCTCTTGGCTCTGGCATCGATGAAGAACGCAGCGAA

Beauveria_bassiana_ATCC_MYA_4886 ACGCGATAAGTAATGTGAATTGCAGAATCCAGTGAATCATCGAATCTTTGAACGCACATT

Fusarium_solani_CBS_119600 ATGCGATAAGTAATGTGAATTGCAAAATTCAGTGAATCATCGAATCTTTGAACGCACATT

Lomentospora_prolificans_JHH_5317 ATGCGATAAGTAATGTGAATTGCAGAATTCAGTGAATCATCGAATCTTTGAACGCACATT

Microascus_brevicaulis_MUCL_40726 ATGCGATAAGTAATGTGAATTGCAGAATTCAGTGAATCATCGAATCTTTGAACGCACATT

Pseudogymnoascus_destructans_ATCC ATGCGATAAGTAATGTGAATTGCAGAATTCAGTGAATCATCGAATCTTTGAACGCACATT

Sarocladium_kiliense_MUCL_9724 ATGCGATAAGTAATGTGAATTGCAGAATTCAGTGAATCATCGAATCTTTGAACGCACATT

Scedosporium_apiospermum_FMR_8619 ATGCGATAAGTAATGTGAATTGCAGAATTCAGTGAATCATCGAATCTTTGAACGCACATT

Scedosporium_aurantiacum_FMR_8630 ATGCGATAAGTAATGTGAATTGCAGAATTCAGTGAATCATCGAATCTTTGAACGCACATT

Sporothrix_schenckii_CBS_359.36 ATGCGATACGTAATGTGAATTGCAGAATTCAGCGAACCATCGAATCTTTGAACGCACATT

Beauveria_bassiana_ATCC_MYA_4886 GCGCCCGCCAGCATTCTGGCGGGCATGCCTGTTCGAGCGTCATTTCAACCCTCGACCTC-

Fusarium_solani_CBS_119600 GCGCCCGCCAGTATTCTGGCGGGCATGCCTGTTCAAGCGTCATTACAACCCTCAGGCCC-

Lomentospora_prolificans_JHH_5317 GCGCCCGGCAGTAATCTGCCGGGCATGCCTGTCCGAGCGTCATTTCAACCCTCGAGCCT-

Microascus_brevicaulis_MUCL_40726 GCGCCCGGCAGCAATCTGCCGGGCATGCCTGTCCGAGCGTCATTTCTTCCCTCGAGCGCG

Pseudogymnoascus_destructans_ATCC GCGCCCCCTGGTATTCCGGGGGGCATGCCTGTCCGAGCGTCATTACAACCCTCAAGCTC-

Sarocladium_kiliense_MUCL_9724 GCGCCCGCCGGCACTCCGGCGGGCATGCCTGTCCGAGCGTCATTTCAACCCTCAGGACC-

Scedosporium_apiospermum_FMR_8619 GCGCCCGGCAGTAATCTGCCGGGCATGCCTGTCCGAGCGTCATTTCAACCCTCGAACCT-

Scedosporium_aurantiacum_FMR_8630 GCGCCCGGCAGTAATCTGCCGGGCATGCCTGTCCGAGCGTCATTTCAACCCTCGAACCT-

Sporothrix_schenckii_CBS_359.36 GCGCCCGCCAGCATTCTGGCGGGCATGCCTGTCCGAGCGTCATTTCCCCCCTCACGCGC-

Beauveria_bassiana_ATCC_MYA_4886 -CCCTTGGG-------------GAGGTCGGCGTTGGGGACCGGCAGCA------------

Fusarium_solani_CBS_119600 -CC-------------------GGGCCTGGCGTTGGGGATCGGCGGAGCCCCCCGTGGGC

Lomentospora_prolificans_JHH_5317 -AGGTTTTTAC----AAGCCCAAGGATCGGTGTTGGGGCGCTACGGTT-GTCATC-TGAC

Microascus_brevicaulis_MUCL_40726 GCTAGCCCTACGGGGCCTGCCGTCGCCCGGTGTTGGGGCTCTACGGGTGGGGCTCGTCCC

Pseudogymnoascus_destructans_ATCC -----------------------AGCTTGGTATTGGGCCCCGCCGA--------------

Sarocladium_kiliense_MUCL_9724 -CCCTTTCGGGGG---------GGACCTGGTGCTGGGGATCAGCGG-----CCTCCGGGC

Scedosporium_apiospermum_FMR_8619 -CCGTTTCCTCAGGGAAGCCC-AGGGTCGGTGTTGGGGCGCTACGGCGAGTCCTCGCGAC

Scedosporium_aurantiacum_FMR_8630 -CTGTTTCC-CAGCGAAGCTC-AGGGTCGGCGTTGGGGCGCTACGGCGAGTCTTCGCGA-

Sporothrix_schenckii_CBS_359.36 -CCCGTTGCGC--------------GCTGGTGTTGGGGCGC------------------C

Beauveria_bassiana_ATCC_MYA_4886 CACCGCC---------GGCCCTGAAATGGAGTGGCGGCCCGTCCGCGG-CGACCTCTGCG

Fusarium_solani_CBS_119600 ACACGCC---------GTCTCCCAAATACAGGGGCGGTCCCGCCGCAG-CTTCCATCGCG

Lomentospora_prolificans_JHH_5317 CGCCGTA---------GGCTCTGAAATACAGTGGCGGTCCCGCCGCGG-CGCCTTCTGCG

Microascus_brevicaulis_MUCL_40726 CCCCGCA---------GTCCCCGAAATGTAGTGGCGGTCCAGCCGCGG-CGCCCCCTGCG

Pseudogymnoascus_destructans_ATCC -CCCGGC---------GGGCCCTAAAGTCAGTGGCGGTGCCGTCC----GGCTCCGAGCG

Sarocladium_kiliense_MUCL_9724 CCCTGTC------------CCCCAAATTGAGTGGCGGTCGCGCCGCAG-CCTCCCCTGCG

Scedosporium_apiospermum_FMR_8619 CCCCGTA---------GGCCCTGAAATACAGTGGCGGTCCCGCCGCGGTTGCCTTCTGCG

Scedosporium_aurantiacum_FMR_8630 CCCCGTA---------GGCCCTGAAATACAGTGGCGGTCCCGCCGCGGTTGCCTTCTGCG

Sporothrix_schenckii_CBS_359.36 CTCCGCCTGGCGGGGGGCCCCCGAAAGCGAGTGGCGGGCCCTGTGGAA-GGCTCCGAGCG

Beauveria_bassiana_ATCC_MYA_4886 CAGTAA-----------TACAGC--TCGCACCGGAACCCCGACGCG-GCCACGCCGTAAA

Fusarium_solani_CBS_119600 TAGTAG---------CTAACACC--TCGCGACTGGAGAGCGGCGCG-GCCACGCCGTAAA

Lomentospora_prolificans_JHH_5317 TAGTAGA--------TTTACAAC--TCGCATT-GGGTCCCGGCGAA-GGCCAGCCGTCAA

Microascus_brevicaulis_MUCL_40726 TAGTAGA-------TCCTACATC--TCGCATC-GGGTCCCGGCGAA-GGCCAGCCGTCGA

Pseudogymnoascus_destructans_ATCC TAGTAA-----------TTCTTC--TCGCTCCGGAGGTCCGGTCGTGTGCTTGCCAGCA-

Sarocladium_kiliense_MUCL_9724 TAGTAG-----------CACACC--TCGCACC-GGAGAGCGGCTCG-GCCACGCCGTGAA

Scedosporium_apiospermum_FMR_8619 TAGTAAG---TCTCTTTTGCAAGC-TCGCATT-GGGTCCCGGCGGA-GGCCTGCCGTCAA

Scedosporium_aurantiacum_FMR_8630 TAGTAAAAGTCTTCTTTTGCAAGCTTCGCATT-GGGTCCCGGCGGA-GGCCTGCCGTCAA

Sporothrix_schenckii_CBS_359.36 CAGTACC-GAACGCATGTTCTCCCCTCGCTCCGGACGCCCCCCAGGCGCCCTGCCGTGAA

Beauveria_bassiana_ATCC_MYA_4886 ----------ACACCCAA----CTTCTGAACG-TT

Fusarium_solani_CBS_119600 ----------ACACCCAA--CTCTTCTGAAG--TT

Lomentospora_prolificans_JHH_5317 ----------ACCCTCTA-----TTCTTATGGTTT

Microascus_brevicaulis_MUCL_40726 ----------ACCTTTTA------TTTCATGGTTT

Pseudogymnoascus_destructans_ATCC ----------ACCCCCAA----TTTTTTCAGG-TT

Sarocladium_kiliense_MUCL_9724 ----------ACCCCCAA----TTTTTTAAGG-TT

Scedosporium_apiospermum_FMR_8619 ----------ACCACCTAACAACTCCAGATGG-TT

Scedosporium_aurantiacum_FMR_8630 ----------ACCACATTATAACTTAAGATGG-TT

Sporothrix_schenckii_CBS_359.36 AACGCGCATGACGCGCAGCTCTTTTTACAAGG-TT

**Supplementary figure 4**. Multiple alignment of ITS regions from *Lomentospora prolificans* and other fungal species used to build the tree in Supplementary Figure 3. The sequences were aligned using Muscle^1^.

**References**

1. Edgar, R.C. MUSCLE: multiple sequence alignment with high accuracy and high throughput. *Nucleic Acids Res* **32**, 1792-1797 (2004).

2. Tamura, K., Stecher, G., Peterson, D., Filipski, A. & Kumar, S. MEGA6: Molecular Evolutionary Genetics Analysis version 6.0. *Mol Biol Evol* **30**, 2725-2729 (2013).
